# Biomarkers of Oxidative Stress, Systemic Inflammation and Thrombosis in Adult Asthmatic Patients Treated with Inhaled Corticosteroids During Exposure to Fine Particulate Matter

**DOI:** 10.3390/jcm14072360

**Published:** 2025-03-29

**Authors:** Warawut Chaiwong, Chalerm Liwsrisakun, Juthamas Inchai, Pilaiporn Duangjit, Chaiwat Bumroongkit, Athavudh Deesomchok, Theerakorn Theerakittikul, Atikun Limsukon, Pattraporn Tajarernmuang, Nutchanok Niyatiwatchanchai, Konlawij Trongtrakul, Chittrawadee Chitchun, Nipon Chattipakorn, Siriporn C. Chattipakorn, Nattayaporn Apaijai, Chaicharn Pothirat

**Affiliations:** 1 Division of Pulmonary, Critical Care, and Allergy, Department of Internal Medicine, Faculty of Medicine, Chiang Mai University, Chiang Mai 50200, Thailand; warawut.chai@cmu.ac.th (W.C.); juthamas.i@cmu.ac.th (J.I.); pilaiporn.th@cmu.ac.th (P.D.); chaiwat.b@cmu.ac.th (C.B.); athavudh.d@cmu.ac.th (A.D.); theerakorn.t@cmu.ac.th (T.T.); atikun.limsukon@cmu.ac.th (A.L.); pattraporn.t@cmu.ac.th (P.T.); nutchanok.n@cmu.ac.th (N.N.); konlawij.tr@cmu.ac.th (K.T.); chittrawadee.chit@cmu.ac.th (C.C.); chaicharn.p@cmu.ac.th (C.P.); 2 Neurophysiology Unit, Cardiac Electrophysiology Research and Training Center, Faculty of Medicine, Chiang Mai University, Chiang Mai 50200, Thailand; nipon.chat@cmu.ac.th (N.C.); siriporn.c@cmu.ac.th (S.C.C.); nattayaporn.a@cmu.ac.th (N.A.); 3 Cardiac Electrophysiology Unit, Department of Physiology, Faculty of Medicine, Chiang Mai University, Chiang Mai 50200, Thailand; 4 Center of Excellence in Cardiac Electrophysiology, Chiang Mai University, Chiang Mai 50200, Thailand; 5 Department of Oral Biology and Diagnostic Sciences, Faculty of Dentistry, Chiang Mai University, Chiang Mai 50200, Thailand

**Keywords:** PM_2.5_, asthma, inhaled corticosteroids, biomarkers, oxidative stress, systemic inflammation, thrombosis

## Abstract

**Background/Objectives:** Inhaled corticosteroids (ICS) affect oxidative stress and systemic inflammation, which might modify the risk of thrombosis in asthmatic patients exposed to particulate matter with an aerodynamic diameter smaller than 2.5 microns (PM_2.5_). Therefore, we aim to know the systemic biomarkers of oxidative stress, inflammation, and coagulation in ICS-treated, well-controlled adult asthmatic patients after exposure to PM_2.5_. **Methods:** This study was conducted to compare urinary biomarkers of oxidative stress, i.e., 8-hydroxydeoxyguanosine (8-OHdG), and blood biomarkers of inflammation and hypercoagulation, i.e., complete blood count (CBC), high-sensitivity C-reactive protein (hsCRP), fibrinogen, D-dimer, tumor necrosis factor-alpha (TNF-α), and interleukins (IL-6 and IL-8), between well-controlled adult asthmatic patients and healthy controls in low and high-pollution periods. **Results:** Forty-one ICS-controlled asthmatic patients and twenty controls were included. Urinary 8-OHdG, white blood cells and differential counts, platelets count, hsCRP, IL-6, and IL-8 in the asthma group were not significantly higher than controls during the same period. The D-dimer level of the asthma patients was significantly higher than the controls (*p* < 0.05). The median level of TNF-α levels during the pollution period in asthma patients was significantly higher than the non-pollution period with levels of 14.3 (9.3, 27.4) and 11.3 (7.8, 21.1) pg/mL, *p* = 0.041, respectively. **Conclusions:** During exposure to PM_2.5_, serum TNF-α was increased while the other markers of oxidative stress and inflammation were not high in ICS-treated asthma. ICS might mitigate PM_2.5_-induced systemic oxidative stress, inflammation, and hypercoagulation in asthma.

## 1. Introduction

Air pollution, particularly fine particulate matter (particulate matter with an aerodynamic diameter smaller than 2.5 microns, PM_2.5_), is a growing problem in global health. Chiang Mai province is located in northern Thailand, where the annual smog season usually starts in January and ends in April, with the peak level of daily PM_2.5_ in March [1]. This period of the year is the dry season when the burning of forests and cornfields occurs annually. PM_2.5_ has been proven to be associated with the development of diseases, the worsening of underlying diseases, and increased mortality, particularly in the cardiopulmonary system [2]. Asthma and thrombotic-associated cardiovascular diseases are two common diseases that can be coincidental and share common risk factors, including exposure to air pollution. Inflammation and oxidative stress were the major mechanisms of PM_2.5_ impact on both diseases [3,4]. In asthma, PM_2.5_ was associated with more symptoms, worsening of control [5], acute exacerbations [6], emergency room visits [7], hospitalization, and increased mortality [8].

PM_2.5_ was shown to be associated with thromboembolic effects on both the arterial and venous sides [9,10]. Mechanisms of PM_2.5_-induced thrombogenesis were proposed to be associated with an imbalance between oxidant/anti-oxidant, high inflammatory cytokine, e.g., interleukin-6 (IL-6), activation of platelet function, stimulation of hemostasis, and decrease in fibrinolysis [11]. Plaque rupture, the major cause of acute coronary syndrome, could be triggered by inflammation [12] and could be evidenced on the same day of acute PM_2.5_ exposure [9].

**Figure 1:**
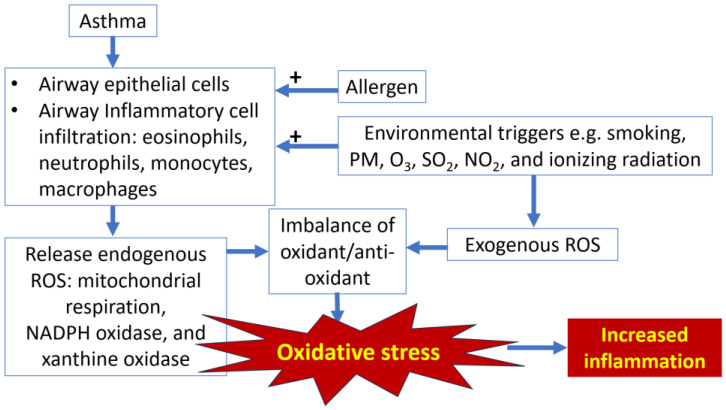
Link between oxidative stress and airway inflammation in asthma. NADPH, nicotinamide adenine dinucleotide phosphate; NO_2_, nitrogen dioxide; O_3_, ozone; PM, particulate matter; ROS, reactive oxygen species; SO_2_, sulfur dioxide.

Asthma is a heterogenous chronic inflammatory airway disease characterized by variation in respiratory symptoms and lung function of airflow obstruction over time. It is a common health problem affecting up to 300 million people worldwide. The impact of asthma is not only developing respiratory symptoms but also causes poor quality of life, absence from workplace and school, emergency room visits, hospitalization, and death [13]. An imbalance between oxidants and antioxidants due to excessive release of oxidants, mainly reactive oxygen species (ROS), either endogenous source from several inflammatory cells or exogenous source from environmental triggers, e.g., PM, smoking, toxic gas, and radiation, results in oxidative stress, which plays an important role in the pathogenesis of airway inflammation in asthma (Figure 1) [14]. Interestingly, asthma itself was a risk factor for acute cardiovascular thromboembolic events. In a meta-analysis, asthma was associated with coronary artery disease with an odds ratio of 1.29 [1.13, 1.46], *p* = 0.001 [15]. Both immune and inflammatory cells, as well as their products involved in asthma pathogenesis, such as mast cells and their cytokines and chemokines in conjunction with cholesterol, could induce atherosclerotic plaque formation on the arterial wall and finally trigger plaque rupture, thrombus and clot formation, and acute coronary syndrome, respectively [16]. Moreover, asthma was a risk factor for the development of pulmonary embolism, particularly severe asthma [17]. Besides airway inflammation, the evidence of systemic inflammation was demonstrated in patients with asthma [18], particularly in persistent asthma [19]. Systemic inflammation might explain the risk of thrombosis in asthma. Inhaled corticosteroids (ICS), the key treatment of asthma, not only suppressed airway inflammation but also lowered the systemic biomarkers such as high-sensitivity C-reactive protein (hsCRP) [20].

Therefore, it is of interest to know the systemic biomarkers of oxidative stress, inflammation, and coagulation in well-controlled asthma treated with ICS after exposure to PM_2.5_.

## 2. Materials and Methods

### 2.1. Study Design and Population

A prospective observational study was conducted in two periods: the first was 20–24 March 2023 (pollution period), and the second was 7–11 August 2023 (non-pollution period). Adult well-controlled asthmatic patients defined by the Asthma Control Test (ACT) ≥ 20 [21], from the Chest Clinic, Division of Pulmonary Critical Care, and Allergy, the Department of Internal Medicine, Faculty of Medicine, Chiang Mai University, Chiang Mai, Thailand, were invited to the study. The inclusion criteria were pulmonologist-diagnosed asthmatic patients, according to the Global Initiative for Asthma (GINA) guidelines [13], who were more than 20 years old. The exclusion criteria were severe asthma requiring systemic steroids or any biologics therapy; uncontrolled underlying diseases with associated systemic inflammation including hypertension, diabetes, dyslipidemia, and BMI > 30 kg/m^2^; current diagnosis of other active respiratory diseases including chronic obstructive pulmonary disease, bronchiectasis, and interstitial lung disease, pregnancy, active cancer, prior three-month history of acute thrombosis, i.e., acute coronary syndrome, stroke, peripheral vascular disease or venous thromboembolism, and current smoking or smoking cessation for less than six months. Fifty-six asthma patients were screened, and forty-three of them agreed to be enrolled in our study. Twenty-one healthy non-asthmatic control subjects, with age- and sex-matching with the patients, were also enrolled for comparison. The control group had to be non-smoking or have ceased smoking for at least six months before enrollment, controlled underlying diseases, non-pregnancy, no prior history of arterial and venous thrombosis, and non-obese [body mass index (BMI) < 30 kg/m^2^]. The subjects in both groups were also excluded from the study if they had a two-week history of either infection, trauma, or operation before blood samplings. This study was approved by the Research Ethics Committee, Faculty of Medicine, Chiang Mai University (Study code: MED-2565-009244, date of approval: 28 October 2022), and filed under the Clinical Trials Registry (Study ID: TCTR20221118003, date of approval: 18 November 2022). Informed consent was obtained from all subjects before enrollment.

Baseline demographics, including age, sex, BMI, level of education, smoking status, underlying diseases, and regularly used medications for both asthma and non-asthma, were recorded during the three months before enrollment. The ICS prescribed for our patients included budesonide, fluticasone furoate, and fluticasone propionate. The doses of ICS in our study were classified as low, medium, or high dose according to the dose equivalence in the 2024 GINA guidelines [13]. Air pollution protection, including home air purifier use and duration of application per day, as well as the outdoor use of N-95 masks during the pollution period, were also recorded. The outcomes of this study were the levels of various blood biomarkers of inflammation and hypercoagulation, i.e., IL-6, IL-8, tumor necrotic factor-alpha (TNF-α), hsCRP, fibrinogen, D-dimer and complete blood count (CBC), and urinary biomarker of oxidative stress, i.e., 8-hydroxy-2′-deoxyguanosine (8-OHdG) during the pollution and non-pollution period in comparison with the healthy subjects.

### 2.2. Measurements of Air Pollutants (PM_2.5_) and Meteorological Parameters

The levels of daily PM_2.5_ were obtained from the sampling stations of the Pollution Control Department, the Ministry of National Resources and Environment of Thailand located in municipal areas of the Muang Chiang Mai District, Chiang Mai Province, Thailand. The monthly average level of PM_2.5_ was calculated from the daily average level of PM_2.5_ in the study months. We used the 2021 WHO standard level of daily PM_2.5_ of 15 µg/m^3^ to determine the high and low monthly averages of PM_2.5_ [2]. The data on meteorology, including daily temperature and humidity, were obtained from the Northern Meteorology Center, located in the municipal area of Muang Chiang Mai District, Chiang Mai, Thailand.

### 2.3. Measurement of Systemic Inflammatory and Hypercoagulable Biomarkers

#### 2.3.1. Complete Blood Count (CBC), High Sensitivity C-Reactive Protein (hsCRP), Fibrinogen and D-Dimer

For CBC, 2 mL of blood was collected in the EDTA tube for flow cytometry (Sysmex XN-9000, Bangkok, Thailand Co., Ltd., Bangkok, Thailand). Four ml of blood were kept in a lithium heparin tube before being sent for assessment of hsCRP using an immunoturbid assay (Cobas^®^ pro-C503, Baku, Azerbaijan). To determine fibrinogen and D-dimer, we collected 2.0 mL of blood in the sodium citrate tube before the measurement of both biomarkers by using a clotting immunoturbidimetric assay (Sysmex CS-2500, Bangkok, Thailand).

#### 2.3.2. Determination of Urine 8-Hydroxydeoxyguanosine (8-OHdG) Levels

Spot urine with a volume of 10–20 mL was collected on the morning of the visit date of the subjects. Competitive ELISA was used to detect urine 8-OHdG levels (Invitrogen, Waltham, MA, USA, Catalog no. EEL004). Results were obtained from the absorbance values of 8-OHdG according to the manufacturer’s instructions.

#### 2.3.3. Determination of Serum Tumor Necrosis Factor-Alpha (TNF-α) and Interleukins (IL-6 and IL-8)

Blood was collected in a clot activator vacuum tube, and serum was obtained by centrifugation of the clot at 3000 rpm for ten minutes before immediately being stored at −85 °C. Serum levels of TNF-α, IL-6, and IL-8 were detected using commercial solid-phase sandwich enzyme-linked immunosorbent assay (ELISA) kits (Invitrogen, Waltham, MA, USA; TNF-α Catalog no. BMS223HS, IL-6 Catalog no. BMS213-2HS, IL-8 Catalog no. # KHC0084). Results were obtained from the absorbance values of TNF-α, IL-6, and IL-8 according to the manufacturer’s instructions. Biomarker assays were conducted within five months of the initial sample collection. Each measurement was performed in duplication to ensure accuracy and reliability.

### 2.4. Sample Size Estimation

The sample size calculation was based on our hypothesis. We estimated the mean and standard deviation (SD) of D-dimer in the pollution period between asthma and healthy subjects were 300 ± 180 ng/mL and 180 ± 120 ng/mL, respectively. Therefore, we needed to study 40 asthma subjects and 20 healthy subjects to be able to reject the null hypothesis that the population means of asthma and healthy subject groups were equal with probability (power) 0.8. The Type I error probability associated with the test of this null hypothesis was 0.05.

### 2.5. Statistical Analysis

Results for continuous data were shown as mean ± standard deviation (SD) or median and interquartile range (IQR) according to their normal contribution. Results for categorical variables were expressed as numbers and percentages (%). Different values of baseline characteristics, hematological data, biomarkers of oxidative stress, inflammation, and thrombosis between asthma and healthy controls were determined using independent *t*-test or Mann–Whitney U-test for parametric and non-parametric data, respectively. Fisher’s exact test was used for the comparison of category data between groups. Pollutant data between low and high pollution periods were analyzed using an independent sample *t*-test. A paired sample *t*-test or Wilcoxon sign rank test was used for the comparison of hematological data, biomarkers of inflammation, and thrombosis between the non-pollution and pollution periods in asthma and healthy controls according to the distributions of data. No adjustments for multiple comparisons were made for multiple biomarkers. Thus, the *p*-values that were presented were unadjusted. Statistical significance was set at a *p*-value < 0.05. All statistical analyses were performed using STATA version 16 (StataCorp, College Station, TX, USA).

## 3. Results

**Figure 2:**
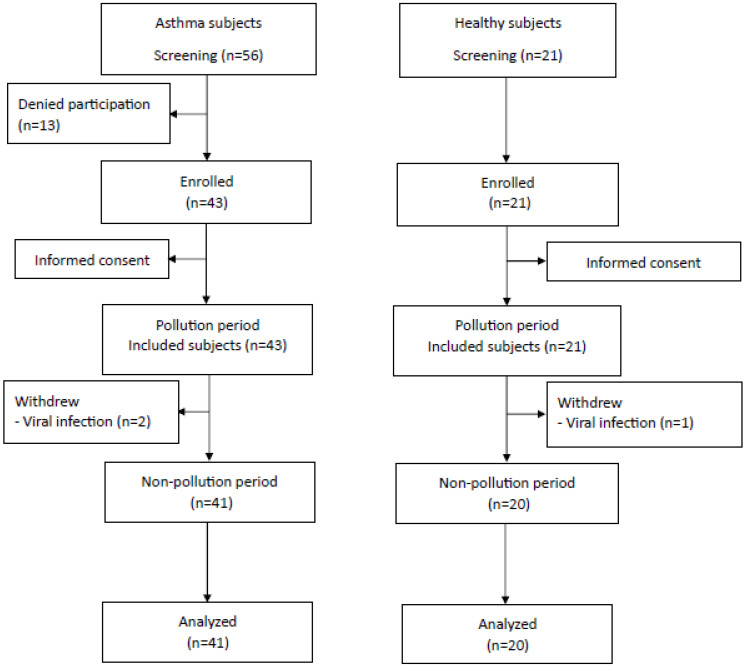
Study Flow.

Sixty-four subjects, 43 of whom had asthma and 21 of whom were healthy control subjects, were enrolled in our study. Two patients in the asthma group and one participant in the control group were excluded from the study because they reported a history of viral infection before blood sampling in either of the two study periods. Therefore, 41 asthmatic patients and 20 control participants remained in the study analysis as the study flow shown in Figure 2. Thirty-nine (92.86%) asthmatic patients in our study had onset of disease after the age of 20. The baseline characteristics of both groups were not different except for the lower use of statins and a lower rate of wearing of N-95 masks during the pollution period in the asthma group, as shown in Table 1. All patients complied with all asthma medications used.

The daily average level of PM_2.5_ and meteorology data for both study periods are displayed in Table 2. The level of PM_2.5_ and temperature were significantly higher in the pollution period, while the relative humidity was significantly lower.

The laboratory findings are shown in Table 3. All parameters of CBC and the levels of fibrinogen and hsCRP did not show any significant difference between the two study periods within both groups and between the asthma group and healthy control, except for the higher blood eosinophil counts (BECs) in the asthma group. The BECs in the asthma group were indifferent between the 2 periods. In contrast, the median levels of D-dimer in asthmatic patients were higher than the controls in each period with levels of 324 (221.0, 484.0) vs. 196.5 (163.0, 329.5) ng/mL, *p* < 0.05, in the non-pollution period and 280.0 (210.5, 426.0) vs. 203.0 (164.0, 263.5) ng/mL, *p* < 0.05, in the pollution period. Levels of D-dimer were indistinguishable between the two periods within the asthma group. When using the age-adjusted cut-off level of higher than 500 ng/mL for age ≤ 50 years and 10 times of age for age > 50 years to define abnormality [22], 9 (22%) and 8 (19.5%) asthmatic patients had high D-dimer in the non-pollution and pollution period, respectively. No one in the healthy group had D-dimer above this threshold in any phase.

For the biomarkers of oxidative stress, systemic inflammation, and hypercoagulation, the urinary 8-OHdG levels were not different either within or between groups in both seasons. Accordantly, the levels of IL-6 in the asthmatic group were not higher than the control group in both periods. However, within the control group, the IL-6 was significantly higher in the non-pollution timing than the pollution timing. Conversely, the IL-8 level in the asthma group was significantly lower than in the control group during the smog season. There were no differences within each group when comparing the two study periods. For TNF-α, its level in the asthmatic group was significantly higher, *p* = 0.041, during the high pollution period than the low pollution period with a level of 14.3 (9.3, 27.4) and 11.3 (7.8, 21.1) pg/mL, respectively.

## 4. Discussion

People in Chiang Mai have suffered from high levels of PM_2.5_ for more than 20 years. The monthly average of daily PM_2.5_ levels in the hot and dry months in our study was significantly higher than the non-pollution period and much higher than the 15 µg/m^3^ of 2021 WHO daily PM_2.5_ standard [2]. This high level of air pollution affected the health of the population, particularly the people at risk, e.g., asthma patients. The reports of the epidemiologic link between asthma and thromboembolic cardiovascular diseases, which were aggravated by PM_2.5_, ignited our curiosity to see the biomarkers of inflammation and thrombosis in well-controlled asthmatic patients after exposure to PM_2.5_.

At the cellular level, alveolar macrophage and airway epithelial cells were involved in PM_2.5_-induced oxidative stress and inflammation, which stimulated many kinds of inflammatory cells, particularly neutrophils [3,23]. Urinary 8-OHdG was the hallmark of oxidative stress-induced DNA damage, which could be found in association with PM_2.5_ exposure [24]. Besides, oxidative stress was associated with airway inflammation in asthma, particularly in uncontrolled asthma [25]. Our result could not demonstrate this relation both in normal subjects and asthmatic patients, which might be due to the treatment effects of both groups. In the asthma group, all patients were well-controlled by ICS plus other controllers according to the level of severity. The anti-inflammatory effects of ICS might result in antioxidant properties and a reduction in oxidative stress [26]. In the control group, most of them had taken statins and antihypertensives for the treatment of their underlying diseases. Both groups of medications were demonstrated to have antioxidant effects [27,28], which might be the cause of the indifferent urinary markers of oxidative stress in our study.

When asthmatic patients are exposed to air pollution, PM_2.5_ could induce oxidative stress directly through alveolar macrophages, releasing many proinflammatory cytokines, including TNF-α, IL-6, and IL-8 [24,29]. After release from alveolar macrophage, TNF-α stimulates T-helper type 1 (T1) cells to release more cytokines, including TNF-α, which are associated with neutrophilic inflammation [30]. Our study showed that TNF-α was the only cytokine that significantly increased during the high-PM_2.5_ exposure in comparison with the period of low-PM_2.5_ exposure in the asthma group. TNF-α was one of the inflammatory biomarkers of T-helper type 2-low (T2-low) pheno-endotype of severe neutrophilic asthma in which ICS failed to suppress airway inflammation and resulted in ICS-resistant severe asthma [31]. Berry et al. showed that patients with steroid-resistant asthma had significantly higher activity of systemic TNF-α than steroid-sensitive asthma patients and controls [32]. Neutrophilic airway inflammation from PM_2.5_ might explain why the level of TNF-α was significantly higher despite treatment with ICS in asthmatic patients during the pollution period in our study.

IL-6 is one of the T1 cytokines involved in neutrophilic airway inflammation [30]. High IL-6 was a risk factor for poor pulmonary function and acute exacerbation in asthma [33] and the development of acute myocardial infarction [34]. One of the mechanisms of IL-6 in asthma severity and poor response to bronchodilator is via vascular endothelial growth factor (VEGF) [35]. An increment in IL-6 [36] and VEGF [37] in response to PM_2.5_ was demonstrated. However, this result was inconsistent because Kim et al. did not find an association between PM_2.5_ and increased IL-6 biomarker [24]. In our study, the IL-6 level during the pollution period was insignificantly higher than the low-pollution period in patients with asthma and not different from healthy control in both seasons. The explanation for this finding might be from the evidence that steroids could inhibit TNF-α induced release of IL-6 from human epithelial cells [38]. Interestingly, the IL-6 level during the low PM_2.5_ period was significantly higher than the period of high PM_2.5_ in healthy controls. We did not know the exact causes of this high IL-6 level. However, IL-6 was not only associated with acute inflammation but could also be found in elevation in various acute and chronic conditions, including trauma, surgery, malignancy, cardiovascular diseases, obesity, and other metabolic diseases [39,40]. Additionally, even exercise could induce the elevation of IL-6 [41]. Furthermore, one of the functions of IL-6 was the regulation of the synthesis of other inflammatory biomarkers, including CRP and fibrinogen [42]. The unexpectedly high IL-6 in healthy adults during the non-pollution period without an increase in hsCRP and fibrinogen levels at the same time would suggest that the level of IL-6 was falsely high.

IL-8 is the neutrophil chemotactic factor released from many cells, including macrophage, airway epithelial cells, and airway smooth muscle cells [43,44]. Our study was consistent with a meta-analysis done by Tang et al., which showed that the level of IL-8 was not significantly high in response to PM_2.5_ [45]. Contradictory, the level of IL-8 was not even increased or normal in our study; it became significantly lower in asthma patients than the healthy participants during the high pollution season. This phenomenon could be explained by the effects of ICS therapy on the inhibition of IL-8 release from airway smooth muscle cells [44]. Moreover, combined ICS and beta2-agonists potentiated this inhibition, which might be attributable to the synergistic anti-inflammatory effects of these two medications in vitro [44]. In vivo studies, combined ICS and long-acting beta2-agonist (LABA) could reduce airway inflammatory cells and IL-8 in asthma patients [46]. All patients in our study received ICS plus LABA for controlling their asthma, which would be attributable to the low IL-8 in this study.

Biomarkers of systemic inflammation, i.e., hsCRP, fibrinogen, and platelets count, were not increased in our study. High-sensitivity CRP is the marker of inflammation in both asthma and cardiovascular disease (CVD). In asthma, hs-CRP was shown to be associated with the severity of asthma and asthma control [47]. A high hsCRP level also significantly predicted the risk of CVD [48]. Although our study demonstrated that during the pollution period, the level of hs-CRP in asthma patients was higher than during the non-pollution period and healthy control, it was not statistically significant. Fibrinogen, the marker of acute phase reaction, pro-inflammation, and hypercoagulation, was associated with severe asthma, more airflow obstruction, more requirements of controller medications, more exacerbations, and higher rates of various cardiovascular comorbid diseases [49]. Our finding on fibrinogen level was in contrast to a meta-analysis, which demonstrated an association between PM_2.5_ and high fibrinogen levels in healthy subjects and patients with cardiopulmonary diseases [36]. Platelets are the blood cells that are involved in the process of hemostasis and thrombosis, particularly on the arterial side. The numbers of platelets in response to PM_2.5_ exposure were reported to be variable in many studies. However, our study allied with a meta-analysis, which showed an insignificant relation between PM_2.5_ and the number of platelets in circulation [50]. The failure to increment in hsCRP, fibrinogen, and platelets number in our study might be from ICS-inhibited IL-6 release because IL-6 acted on inducing many coagulating factors and activation of platelets [51].

D-dimer, the product of fibrin degradation as the result of fibrin formation and fibrinolysis, was found to be higher in asthma patients than in healthy controls [52]. This finding was compatible with the previous study, which showed the same result [52]. In addition, Tattersall et al. showed that the level of D-dimer increased correspondingly to the severity of asthma, despite regular ICS treatment [19]. Around one-fifth of our asthma patients had an elevation of D-dimer levels above the normal cut-off value and showed no difference between smog and non-smog seasons. This finding might be interpreted as D-dimer increased because of asthma itself, not the effect of PM_2.5_, and supported the evidence that asthma had a high risk of thrombogenesis. Our finding on the significantly higher level of D-dimer in asthmatic patients than in healthy controls, even during the non-pollution period, is interesting. It means that ICS may not completely suppress inflammation-induced thrombosis in all patients with asthma, and they still have the risk of atherosclerotic cardiovascular disease (ASCVD) despite being treated with ICS. This phenomenon can be explained by asthma heterogeneity because asthma is a complex disease composed of many phenotypes and endotypes, which are variable in cellular and cytokine involvement, as well as the response to ICS. For example, non-eosinophilic, T2-low asthma may be associated with neutrophilic inflammation and resistance to ICS treatment [53]. The significance of high D-dimer versus low D-dimer asthmatic patients for the future risk of ASCVD development needs further studies. The effects of PM_2.5_ on D-dimer levels from previous studies were inconsistent. Although our result harmonized with the previous study, which demonstrated no correlation between D-dimer and PM_2.5_ exposure [50], another study showed a significant relation [54]. Differences in the age and ethnicity of the study population might explain these dissociated findings. In addition, Zhang et al. revealed that exposure to different sources and different components of PM_2.5_ could make some variable D-dimer responses [55].

Besides the impact of ICS use on the outcomes of our study, most of our subjects also received statins to prevent CVD. Thomson et al. showed that adding oral statins to ICS could lower sputum inflammatory biomarkers than ICS alone for the treatment of smoking patients with asthma [56]. Additionally, a meta-analysis revealed that statins alone or in combination with ICS could significantly reduce the sputum IL-6 and serum hs-CRP levels [57]. These findings confirmed the beneficial effects of pharmacological control of the underlying diseases in reducing inflammation from PM_2.5_ exposure. Besides statins, some of our patients had a history of using NAC, which has antioxidant and anti-inflammatory activities. In an animal model, Lin et al. showed that NAC could suppress oxidative lung injury after exposure to PM_2.5_ [58]. Although it might affect the result on 8-OHdG level, only 5 (12.2%) of our asthmatic patients reported using this medication. Moreover, all of them used it for mucolytic purposes in an as-needed manner, not regularly for long-term use for expecting antioxidant properties. Therefore, its effect on the biomarkers in our study was expected to be trivial.

Our study strengthened the findings from previous studies, which showed that not all asthmatic patients increased the risk of thrombosis. A study done by Bang et al. showed that only asthma patients without good control, requiring extra or emergency visits and hospitalization, were at significant risk of acute MI [59]. Moreover, evidence showed that the use of ICS was associated with a lower risk of acute myocardial infarction [60] and lower cardiovascular and overall mortality in patients with asthma [61].

The strength of this study was the study on biomarkers of oxidative stress, inflammation, and hypercoagulability after exposure to PM_2.5_ that might intensify the risk of thrombotic events in asthma patients. The results of this study directly supported the bridging between these pathogenesis and epidemiologic evidence of thrombosis in asthma and the possible mitigated effects of medications used for the underlying diseases, including ICS, antihypertensive, and statins. However, our study had some limitations. Firstly, we could not control some confounders, which might affect the results of the study, e.g., the use of air purifiers, wearing N-95 masks, and medication use, including NAC. Secondly, most of our subjects were female. There was evidence that showed that gender was one of the factors determining the level of some biomarkers [62] and the risk of coronary heart disease in asthmatic patients [15]. The higher ratio of female patients might affect the biomarker responses in our study. Thirdly, we had no asthma patients who did not use ICS for comparison. However, the inclusion of this group of patients in the study might be unethical. Fourthly, the potential confounding influence of statins use was not analyzed in our study. Thus, the effect of statins on biomarkers of oxidative stress, inflammation, and hypercoagulability after exposure to PM_2.5_ in asthma should be explored in a future study.

## 5. Conclusions

Our study demonstrated that PM_2.5_ exposure could induce higher serum TNF-α, which supported the evidence of inflammation and hypercoagulation in terms of biomarkers in response to PM_2.5_ inhalation in patients with asthma. Meanwhile, the failure to increase in IL-6 and IL-8 might be from the effects of ICS treatment. The non-significant effects of PM_2.5_ on hsCRP, fibrinogen, and platelets might be from the suppression of IL-6 expression by ICS as well. The index of PM_2.5_-induced oxidative stress, urinary 8-OHdG, also showed no significant difference, which might be from ICS and the high rate of statin use in our asthmatic patients. The significantly higher D-dimer levels of asthmatic patients, irrespective of PM_2.5_ exposure, might support the risk of thrombosis in asthmatic patients. Our biomarker study encouraged the benefits of regular treatment with ICS in asthmatic patients, particularly if a prothrombotic state is evidenced during a high PM_2.5_ period.

## Figures and Tables

**Table 1:** Demographic Data of Study Participants and Information on Exposure to Polluted Air in Asthma Patients and Healthy Controls.

Variables	Asthma (n = 41)	Healthy Controls (n = 20)	*p*-Value
Age (years)	55.7 ± 12.1	55.9 ± 8.3	0.936
Female sex, n (%)	29 (70.7)	13 (65.0)	0.770
Height (cm)	156.2 ± 7.8	157.5 ± 6.9	0.519
Body weight (kg)	63.4 ± 14.8	64.1 ± 10.4	0.859
BMI (kg/m^2^)	25.9 ± 5.3	25.7 ± 3.0	0.897
Age of asthma onset (years)	38.7 ± 14.4	N.A.	
Smoking status, n (%)			1.000
Non-smoker	37 (90.2)	19 (95.0)	
Current-smoker	0 (0.0)	0 (0.0)	
Ex-smoker	4 (9.8)	1 (5.0)	
Education level			0.558
Primary	8 (19.5)	2 (10.0)	
Secondary	14 (34.1)	9 (45.0)	
Bachelor’s degree or higher	19 (46.3)	9 (45.0)	
Underlying disease			0.060
No	21 (51.2)	3 (15.0)	
Hypertension	3 (7.3)	1 (5.0)	
Dyslipidemia	5 (12.2)	5 (25.0)	
Hypertension + Dyslipidemia	7 (17.1)	8 (40.0)	
DM + Dyslipidemia	2 (4.9)	0 (0.0)	
Hypertension + DM + Dyslipidemia	3 (7.3)	3 (15.0)	
Inhaled medication used			
ICS + LABA	39 (95.1)	N.A.	
ICS + LABA + LAMA	2 (4.9)	N.A.	
ICS (dose)			
Low	25 (61.0)	N.A.	
Medium	15 (36.6)	N.A.	
High	1 (2.4)	N.A.	
N-acetyl cysteine (NAC) use	5 (12.2)	N.A.	
Statins use	17 (41.5)	16 (80.0)	0.006
Antihypertensives	13 (31.7)	12 (60)	0.052
Information on Exposure to Polluted Air in Pollution Period			
Use pollution protection, e.g., N-95 mask (yes)	4 (9.8)	10 (50.0)	0.001
Have an air purifier at home (yes)	20 (48.8)	12 (60.0)	0.430
Duration of air purifier use (hours/day) (median, IQR)	8.0 (6.5, 10.5)	10.0 (8.0, 18.8)	0.248

Notes: Results are expressed as mean ± SD or n (%) if not specified. Abbreviations: BMI, body mass index; DM, diabetes mellitus: ICS, inhaled corticosteroids; LABA, long-acting beta2-agonists; LAMA, long-acting muscarinic antagonists; N.A., not available.

**Table 2:** Monthly Average of Daily Average PM_2.5_, Temperature, and Humidity Between the High Pollution Period (March 2023) and the Low Pollution Period (August 2023).

Variables	Non-Pollution Period	Pollution Period	*p*-Value
PM_2.5_ (µg/m^3^)	12.7 ± 2.5	71.9 ± 22.9	<0.001
Temperature (Celsius)	25.1 ± 1.4	27.5 ± 1.6	<0.001
Humidity (%)	76.8 ± 10.1	57.2 ± 6.9	<0.001

Note: Results are expressed as mean ± SD. Abbreviations: PM_2.5_, particulate matters with a diameter of smaller than 2.5 microns.

**Table 3:** CBC and Biomarkers of Inflammation and Thrombosis between the Non-Pollution and Pollution Periods in Asthma and Healthy Controls.

Variables	Asthma (n = 41)			Healthy Controls (n = 20)		
	Non-Pollution Period	Pollution Period	*p*-Value	Non-Pollution Period	Pollution Period	*p*-Value
**CBC** (mean ± SD)						
Hemoglobin (g/dL)	13.2 ± 1.5	13.0 ± 1.4	0.058	13.1 ± 1.5	13.1 ± 1.6	0.936
Hematocrit (%)	40.7 ± 4.3	41.3 ± 4.0	0.061	40.5 ± 3.1	41.1 ± 3.8	0.188
White blood cells (×10^3^ cells/mm^3^)	6.7 ± 1.8	7.1 ± 2.6	0.156	6.4 ± 1.4	6.1 ± 1.4	0.406
Neutrophil count (×10^3^ cells/mm^3^)	3.9 (2.9, 4.6)	3.6 (2.8, 5.3)	0.892	3.5 (2.6, 4.6)	3.1 (2.6, 4.1)	0.502
Lymphocyte count (×10^3^ cells/mm^3^)	1.9 ± 0.6	2.0 ± 0.6	0.427	2.1 ± 0.4	2.1 ± 0.5	0.823
Eosinophil count (cells/mm^3^)	276.8 * (149.4, 459.9)	267.5 ** (140.1, 406.9)	0.564	134.9 (75.6, 223.8)	153.0 (89.6, 225.6)	0.852
Platelet count (×10^3^ platelets/mm^3^)	279.5 ± 59.6	279.8 ± 66.0	0.950	264.6 ± 51.1	258.7 ± 46.7	0.384
**Fibrinogen (mg/dL)**	293.0 (253.5, 344.2)	312.3 (255.8, 356.2)	0.346	280.0 (234.0, 329.0)	288.2 (245.0, 332.9)	0.251
**hsCRP (mg/L)**	1.1 (0.7, 2.9)	1.6 (0.7, 4.1)	0.162	0.9 (0.5, 2.9)	1.3 (0.7, 2.5)	0.605
**D-dimer (ng/mL)**	324.0 * (221.0, 484.0)	280.0 ** (210.5, 426.0)	0.712	196.5 (163.0, 329.5)	203.0 (164.0, 263.5)	0.220
**Oxidative stress biomarker**						
8-OHdG (ng/mL)	11.6 (9.5, 13.0)	10.9 (9.5, 13.2)	0.776	11.8 (9.2, 13.4)	11.2 (9.9, 12.9)	0.709
**Inflammatory and thrombosis biomarkers**						
IL-6 (pg/mL)	2.1 (1.3, 4.5)	2.6 (1.5, 5.1)	0.539	4.5 (1.0, 8.8)	2.6 (2.1, 4.5)	0.039
IL-8 (pg/mL)	4.5 (3.5, 6.3)	4.2 (2.9, 5.5) **	0.096	4.6 (3.5, 6.2)	5.9 (4.4, 8.5)	0.057
TNF-α (pg/mL)	11.3 (7.8, 21.1)	14.3 (9.3, 27.4)	0.041	9.4 (7.2, 19.2)	9.9 (7.3, 20.1)	0.455

Notes: Results are expressed as mean ± SD, median (IQR) or n (%); *, *p* < 0.05 for comparison between groups in non-pollution period; **, *p* < 0.05 for comparison between groups in pollution period. Abbreviations: CBC, complete blood count; hsCRP, high sensitivity C-reactive protein; IL-6, interleukin-6; IL-8, interleukin-8; 8-OHdG, 8-hydroxy-2′-deoxyguanosine; TNF-α, tumor necrosis factor α.

## Data Availability

The data that support the findings of this study are available from the corresponding author upon reasonable request.
